# Dynamic Construction Scheme for Virtualization Security Service in Software-Defined Networks

**DOI:** 10.3390/s17040920

**Published:** 2017-04-21

**Authors:** Zhaowen Lin, Dan Tao, Zhenji Wang

**Affiliations:** 1 Network and Information Center, Institute of Network Technology, Beijing University of Posts and Telecommunications, Beijing 100876, China; linzw@bupt.edu.cn; 2 Science and Technology on Information Transmission and Dissemination in Communication Networks Laboratory, Beijing University of Posts and Telecommunications, Beijing 100876, China; 3 National Engineering Laboratory for Mobile Network Security, Beijing University of Posts and Telecommunications, Beijing 100876, China; 4 School of Electronic and Information Engineering, Beijing Jiaotong University, Beijing 100044, China; 14120034@bjtu.edu.cn; 5 Jiangsu High Technology Research Key Laboratory for Wireless Sensor Networks, Nanjing 210003, China

**Keywords:** software defined network, security service, service composition, RETE

## Abstract

For a Software Defined Network (SDN), security is an important factor affecting its large-scale deployment. The existing security solutions for SDN mainly focus on the controller itself, which has to handle all the security protection tasks by using the programmability of the network. This will undoubtedly involve a heavy burden for the controller. More devastatingly, once the controller itself is attacked, the entire network will be paralyzed. Motivated by this, this paper proposes a novel security protection architecture for SDN. We design a security service orchestration center in the control plane of SDN, and this center physically decouples from the SDN controller and constructs SDN security services. We adopt virtualization technology to construct a security meta-function library, and propose a dynamic security service composition construction algorithm based on web service composition technology. The rule-combining method is used to combine security meta-functions to construct security services which meet the requirements of users. Moreover, the RETE algorithm is introduced to improve the efficiency of the rule-combining method. We evaluate our solutions in a realistic scenario based on OpenStack. Substantial experimental results demonstrate the effectiveness of our solutions that contribute to achieve the effective security protection with a small burden of the SDN controller.

## 1. Introduction

Recently, with the rapid development of Internet and network virtualization technology becoming widely used, the traditional network architecture is unable to handle massive network traffic data. Traditional network security protection systems lack unified design and deployment, which leads to the exposure of more and more defects, such as security threats on cyberspace, wide variety of network security, and the lack of unified management interface [1].At the same time, the development of network virtualization technology urgently calls for the innovation of network architecture. Software-defined networking (SDN) is an architecture purporting to be dynamic, manageable, cost-effective, and adaptable, seeking to be suitable for the high-bandwidth, dynamic nature of today’s applications [2]. The SDN architecture is based on decoupling the control plane from the data plane [3]. With its physically distributed but logically centralized controlled networking framework, SDN offers limitless features worthy of investigation.

Due to the increase of sophisticated network attacks, the legacy security services find it difficult to cope with such network attacks in an autonomous manner. SDN has been introduced to make networks more controllable and manageable, and SDN technology promises to autonomously deal with such network attacks in a prompt manner. SDN security has drawn intensified concerns from researchers. Their studies have mainly focused on two aspects: (i) improving the traditional network security using SDN [4]; and (ii) improving SDN security itself [5,6]. The former focuses on how SDN brings new solutions to the traditional network security. The latter pays more attention to security itself in SDN architecture, which is the concern of this paper.

Further, the SDN security problem can be classified into two categories. One is the traditional SDN security problem (traditional network attack), which includes switch security, fake network data, management station security, etc. The other is the special SDN security problem, which includes controller security, interface protocols security, the consistency and validation of flow rule, etc. Traditional network security threats such as malicious data flow attack, table manipulation, application software vulnerabilities, confidentiality and availability threats of data management still occur in the context of SDN. This dependence on the controller will aggravate its burden [7]. Therefore, how to share the burden of controller is an important challenge in SDN security protection, and this is an important issue in this paper.

The main contributions of this paper are summarized as follows. Firstly, we propose a novel security protection architecture for SDN and design a security service orchestration center in the control plane of SDN. This center physically decouples from SDN controller and constructs SDN security services. Secondly, by drawing from the rule-combining method in web service composition, a rule-combining strategy based on expert system is presented to dynamically combine security meta-functions as required. In particular, the RETE algorithm is introduced in order to improve the speed of service composition to satisfy the requirements from massive users.

The remainder of this paper is organized as follows. Section 2 overviews the related work. Section 3 presents a new security service architecture for SDN. Section 4 proposes a dynamic construction scheme for virtualization security service . Emulations and numerical results are given in Section 5 and we conclude this paper in Section 6.

## 2. Related Work

From the available information, we can find that the study on SDN virtualization network security protection is still at an early stage. SDN virtualization security service construction mainly involves three aspects: SDN security, network function virtualization and security service composition, and they respectively provide theory, technology and method support.

### 2.1. SDN Security

There have appeared some study accumulations and achievements on SDN security, such as the vulnerability of controller [6,8,9], the consistency of flow rules [10,11], interface standardization [9,10] and specific security threats [12].

The authors of [6] extended security functions on the basis of the floodlight controller, including security application management, certification service, role-based authorization and security audit, in order to build a new security protection controller named SE-floodlight. The authors of [13] proposed a new SDN security architecture and introduced a resource pool and some key modules (e.g., application management, intrusion tolerance) into the traditional controller for security improvement. The authors of [14] proposed two security architectures for realizing SDN security: Virtualized Security Appliance (VSA) and Software Defined Security (SDS). For the former, security can be embedded into the SDN network by traditional security device virtualization. For the latter, it can separate and reconstruct the control plane and data plane to achieve modularity and reusability. The common drawback of these is that the control plane, which performs security protection, can be handled by the controller alone. It is unavoidable to increase the burden on the controller. Once the controller itself is attacked, the entire network will be paralyzed.

### 2.2. Network Function Virtualization

Research on Network function virtualization (NFV) and SDN cannot be separated from each other. NFV decouples software from hardware to enable network business to be deployed flexibly. However, SDN decouples the control plane from the data plane. NFV technology can be applied to all the network elements (NE) in the network, such as data exchange NE (e.g., Broadband Remote Access Server), traffic analysis equipment (e.g., Deep Packet Inspection), service security equipment (e.g., Service-Level Agreement (SLA) equipment, Content Delivery Network), and security products (e.g., firewall, intrusion detection system) [15]. The construction of SDN virtualization security service depends on NFV technology, namely, we need virtualize all the security meta-functions in the network. The authors of [16] proposed a framework for protecting network resources via SDN-based security services using an Interface to Network Security Functions (I2NSF). The aim was to create a self-governed protection system against network attacks, capable of providing rapid responses to new threats.

### 2.3. Security Service Composition

The process of developing a composite service is called service composition. Security service composition originates from Web service composition. Web service composition is essentially a plug-in or interface composition technology, which allows the definition of increasingly complex applications by progressively aggregating components (security services) at higher levels of abstraction. There exist many mature Web service composition methods. The authors of [17] summarized several common Web service composition methods: static vs. dynamic, model-driven, declarative, automated vs. manual, and context-based. Rule optimization was also involved in security service composition [18]. The authors of [19] optimized the results of the rule-based service composition by using the RETE algorithm and drew a conclusion that RETE algorithm greatly improved the composition efficiency.

## 3. Security Service Architecture

**Figure 1:**
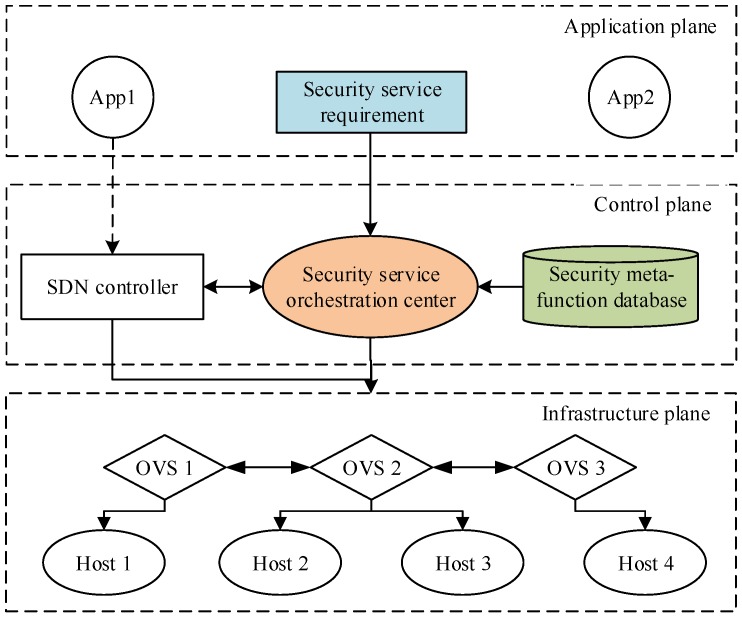
Security protection architecture for SDN.

The security service architecture is shown in Figure 1. This architecture can be divided into three planes: infrastructure plane, control plane and application plane.

The infrastructure plane includes switches, routers and other devices. In this paper, it is mainly made up of Open VSwitch (OVS) and HOST. The application plane is used to control some applications in SDN. Here, the application plane is considered as a security service requirement. In detail, a user can send an application service requirement via a certain application. The control plane is the core of this architecture, and is composed of controllers. In addition, the control plane includes a security service orchestration center which cooperatively performs the construction of virtualization security services in SDN. SDN decouples this center from the controller to relieve its load. This center integrates security services in the traditional network using virtualization technology, which efficiently protects traditional security problems. Moreover, the security service orchestration center is responsible for extracting security meta-functions from the security meta-function library. Hence, how to design security meta-functions is also an important factor to be considered. Some solutions can be described in three aspects: security service requirement description, security service orchestration center, security meta-function.

### 3.1. Security Service Requirement Description

The parsing of security service is to identify the requirement of the security service. This means that the requirement of security service should be described. An effective description language for service requirement helps a machine better understand Web service and thus combine Web service intelligently. Among several service requirement description languages, JavaScript Object Notation (JSON) has unique properties. For example, JSON is a language-independent data format. It derives from JavaScript. Code to generate and parse JSON-format data is available in many programming languages. These properties make JSON an ideal data-interchange language.

Here, we adopt JSON as service requirement description language. The format of a security service requirement can be described as follows:

 

*S* = {“User”: “tom”, “UserID”: “1111”, “GroupID”

: “1002”, “Object”: “web server”, “Address”:

“[192.168.1.1]”

“Request description”:

{“Protection Object Type”: “server”,

“Protection Level”: “1”,

“Protection Functions request”: “SYN, DOS”,

“Protection Performance request”:

{“Width”: “”, “Time-delay”: “”}

}”Result Feedback”: “”}

### 3.2. Security Service Orchestration Center

**Figure 2:**
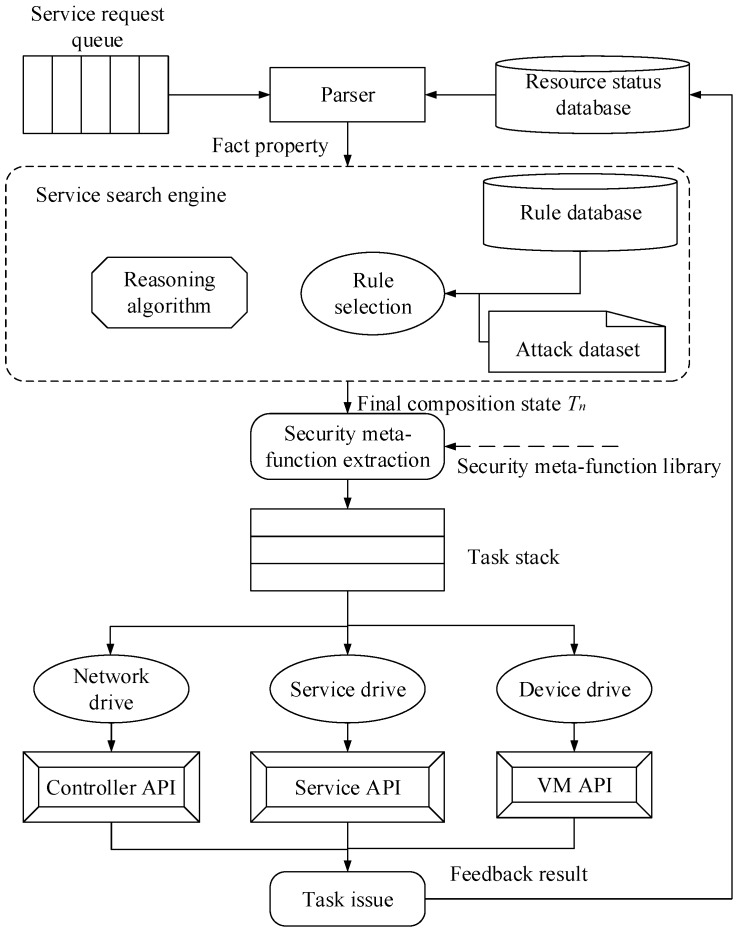
Architecture of the security service orchestration center.

The security service orchestration center is the core part of SDN virtualization security service construction, and includes service request queue, parser, service search engine, database, feature extraction, drive, etc. The architecture of the security service orchestration center is illustrated in Figure 2, which can provide security protection for the SDN controller as well as the whole network.

The kernel of the security service orchestration center is the service search engine, which consists of the reasoning algorithm, rule selection, attack dataset and rule database. The attack dataset and the rule database are constructed according to expert knowledge, and the elements in the attack dataset are the basis of rule selection. The fact property affects the choice of attack elements in the attack dataset, and can be obtained from a requirement description.

The workflow of rule composition based on expert system can be described as following. A user request is sent to the rule composition unit by the parser. After extracting the fact property from a service request, rule composition will match the corresponding attack elements from the attack dataset. The reasoning algorithm sets the initial state, and its corresponding rule will be selected from the rule database. The item of rule strategy is included in a rule, and determines which kind of security meta-function will be used. This strategy is then written into the initial state, and the initial state turns into the next state. The rule searching will go on until the final state. In other words, the process in which the state changes is the one that determines the meta-functions. The security meta-function extracted from the library can be issued and executed by a security agent. Finally, a security logging is recorded in the resource state database and fed back from a security service scheme to the user, and thus completes the whole process of rule composition.

### 3.3. Security Meta-Function

A security service which consists of security meta-functions can be determined by security threats in SDN. The nature of security threats in SDN is as same as that in a traditional network. Security threats come from physical layer, network layer, system, virus, and data transmission. There are three kinds of common security protection approaches in the traditional network, namely, firewall, intrusion detection, anti-virus system [20].

Directly inspired by the security protection ways of the traditional network, we consider firewall and intrusion detection as the two main service types of security protections in SDN. Here, anti-virus will not be considered any longer because of its high demand for real-time updates. Besides the firewall and the intrusion detection mentioned above, SDN itself can complete dynamic flow scheduling service. This service can dynamically specify flow path(s) in situations in which network congestion is caused by a network attack. Hence, flow scheduling is considered as a type of security service in SDN. In addition, the deployment of security service in the virtualization environment requires the support of a Kernel-based Virtual Machine (KVM). So, the KVM service is also considered. In conclusion, there are four types of security services included in this system: firewall, intrusion detection, flow scheduling and KVM.

Different security meta-functions make up different types of security services. Four different types of security services are described below.

- Firewall is a network security system that monitors and controls the incoming and outgoing network traffic based on predetermined security rules [21]. The firewall typically establishes a barrier between a trusted, secure internal network and another outside network.
- Intrusion detection monitors a network or systems for malicious activity or policy violations by collecting network behaviors, security logs, audit data, and other key information in a network system [22].
- SDN flow scheduling is unique, and is attributed to the separation of the control plane and data plane of SDN. Once a certain link (or path) congestion occurs in the network, other optional links can be re-assigned or a black list can be set for a certain link.
- Because the system design is implemented on OpenStack, KVM service class is defined considering that the deployment of some security services might require additional network equipment as a carrier.

In all, four types of security meta-functions included are listed in Table 1.

## 4. Dynamic Security Service Construction Algorithm

The dynamic security service construction algorithm consists of two sub-algorithms: rule-combining algorithm and optimized rule-combining algorithm. The rule-combining algorithm is used to perform rule composition; and the optimized rule-combining algorithm is used to solve the problem of long service response time. In the dynamic security service construction algorithm, the problem of rule based security service composition can be defined as a six-element model $\langle P,F,E,R,T,O\rangle$. The meanings of these elements in this model can be described as follows.

- *P* (service requirement): is described by JSON-format.
- *F* (fact property): is determined by service requirement.
- *E* (attack dataset): corresponds to security service.
- *R* (rule dataset): is the basis of security task execution.
- *T* (composition state): composition state recorded.
- *O* (feedback result): the feedback result of security service composition to a user.

### 4.1. Rule-Combining Algorithm

The rule-combining algorithm is the basis of the security service construction center. The rules need to be designed and described before the rules are combined. Rule description is how to represent expert knowledge in the form that program can handle. For a specific field, we should choose the corresponding rule description which matches the reasoning method. Forward regular reasoning and backward regular reasoning are quite distinct from each other. In detail, forward regular reasoning starts with the available data and uses inference rules to extract more data until a goal is reached. Its disadvantage is that the search time in large-scale rule database has to increase. Backward regular reasoning starts with a list of goals and works backwards from the consequent to the antecedent to see if there is data available that will support any of these consequents. The advantage of backward reasoning is pertinence without searching knowledge which is unrelated to the goal. Considering the actual situation of security service, our system makes use of the forward regular reasoning algorithm.

Some parameters used in the process of forward reasoning are listed in Table 2.

**Figure 3:**
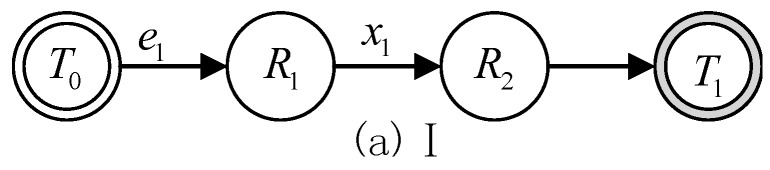
State transition diagram I.

**Figure 4:**
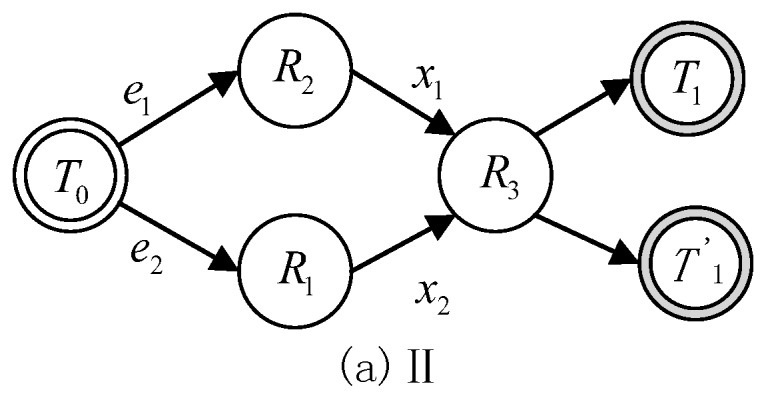
State transition diagram II.

**Figure 5:**
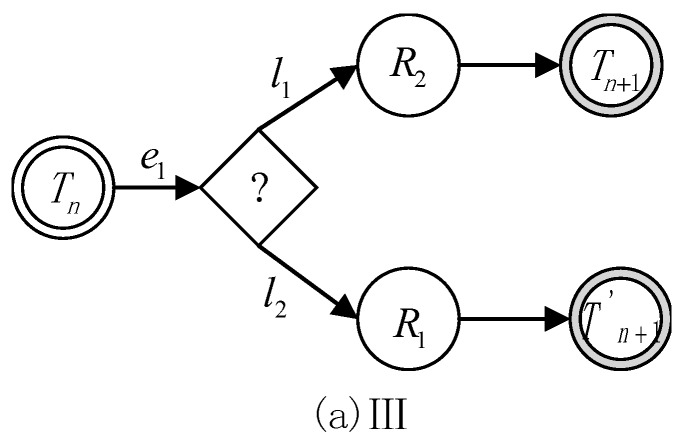
State transition diagram III.

There are three types of basic state transition formulas between the two reasoning states, which are defined as follows. Moreover, their corresponding state transition diagrams are given in Figure 3, Figure 4 and Figure 5.

(1) $$T_{1}=T_{0}\cap \left(R_{1}\cup R_{2}\right),\left(e_{1}\;\&\;R_{1}\overset{x_{1}}{\to }R_{2}\right)$$

(2) $$\left\{\begin{array}{c} T_{1}=T_{0}\cap \left(R_{2}\cup R_{3}\right),\left(e_{1}\;\&\;R_{2}\overset{x_{1}}{\to }R_{3}\right)\\ T_{1}^{\prime }=T_{0}\cap \left(R_{1}\cup R_{3}\right),\left(e_{2}\;\&\;R_{1}\overset{x_{2}}{\to }R_{3}\right) \end{array}\right.$$

(3) $$\left\{\begin{array}{c} T_{n+1}=T_{n}\cap \left(R_{2}\right),\left(e_{1}\;\&\;l_{1}\right)\\ T_{n+1}^{\prime }=T_{n}\cap \left(R_{1}\right),\left(e_{1}\;\&\;l_{2}\right) \end{array}\right.$$

Formula (1) represents a reasoning process that state $T_{0}$ selects rule $R_{1}$ with the condition $e_{1}$. Meanwhile, $R_{1}$ has a rely rule $R_{2}$. After adding these two rules into state $T_{0}$, the reasoning process achieves the state transition from $T_{0}$ to $T_{1}$.

Formula (2) represents a reasoning process such that the initial state $T_{0}$ selects a different rule $R_{i}$ with a different constraint $e_{i}$. However, different $R_{i}$ may have the same rely rule $R_{3}$, which leads to a different state transition $T_{1}$ or $T_{1}^{\prime }$.

Formula (3) represents that state $T_{n}$ may have two kinds of alternative rules with the condition $e_{i}$, but these two rules have different priorities. In this case, the choice of rule can be decided by rule priority $l_{i}$.

**Figure 6:**
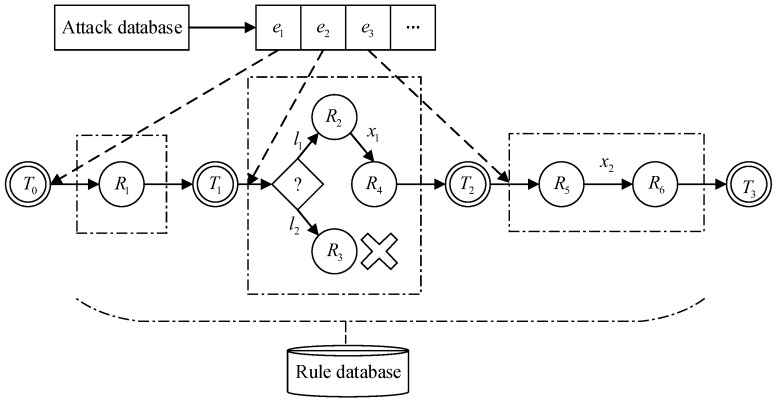
State transition diagram.

These basic state transitions constitute a general state transition (see Figure 6). After the reasoning process mentioned above, the final state $T_{n}$ is obtained. $T_{n}$ can be a choice basis for extracting security meta-functions.

(4) $$T_{n}=T_{0}\cap \left(R_{i}\cup \ldots \cup R_{j}\right),\sum_{i,j=0}^{n}\left(e_{i}\;\&\;l_{i}\;\&\;R_{i}\overset{x_{i}}{\to }R_{j}\right)$$

The description of the expert system based rule-combining algorithm is given in Algorithm 1.
**Algorithm 1** Expert system based rule-combining algorithm.**Input:**
P={name,ID,type,property}.**Output:**
$O=\{O_{1},O_{2},\ldots ,O_{n}\}$.**begin** INIT_REQUEST(P); //initialize queue F.property(P.type); //extract fact property FOR i=0 TO E.number //determine the elements in the attack dataset  IF (F.property IN E.type) THEN   e=E.content;   RETURN;  ELSE   RETURN NULL;  ENDIF ENDFOR INIT_T(T); //initialize state T IF e!=NULL THEN  WHILE(e[i]) DO   IF number(R.AttackName(e[i]))>1 THEN    R=CONFLICT(R.AttackName, l[i]=P.property);   ELSE    R=MATCH(e[i])   ENDIF   T.ADD(R.Strategy); //add state T   WHILE(R.Rely) DO //judge rule rely    T.add(R.Rely.Strategy);    R.Rely=R.Rely.Rely;   ENDWHILE  ENDWHILE ENDIF RETURN *T* //return state T INIT_STACK(); stack.append(T); //add state T into the task stack WHILE(stack!=NULL) DO  TaskPickup(stack.pop()); //execute the task in stack  O.append(Task.state); //feedback task state to result ENDWHILE RETURN *O***end**

### 4.2. Optimized Rule-Combining Algorithm

During the process of SDN security service construction, the increase of the number of users causes the explosive growth of security service requirements. There is no doubt that the rule-combining time will increase greatly. The main reason for this is that for each rule composition, query rules have to be traversed in the database. It is very time-consuming to traverse a database with a great amount of data. This will make service response time too long. To solve this problem, this paper takes the idea from the RETE algorithm in order to optimize the proposed SDN security service construction algorithm.

The RETE algorithm is a pattern matching algorithm for implementing production rule systems. It is used to determine which of the system’s rules should fire based on its data store. The RETE algorithm provides a generalized logical description of an implementation of functionality responsible for matching fact property using rules in a pattern-matching production system. The result consists of one or more conditions and a set of actions which may be undertaken for each complete set of facts that match the conditions. Conditions test fact attributes, including fact type specifiers/identifiers. The RETE algorithm exhibits the following major characteristics [23]:

- It reduces or eliminates certain types of redundancy through the use of node sharing.
- It stores partial matches when performing joins between different fact types. This, in turn, allows production systems to avoid complete re-evaluation of all facts each time changes are made to the production system’s working memory. Instead, the production system needs only to evaluate the changes (deltas) to working memory.
- This allows for efficient removal of memory elements when facts are retracted from working memory.
- This provides a means for many-many matching, an important feature when many or all possible solutions in a search network must be found.

The RETE algorithm can be divided into two parts: rule complication and runtime execution. The compiled result of RETE algorithm is a directed acyclic graph (RETE network) that represents higher-level rule sets. The RETE network includes root node, type node, α node, β node and action node.

- α network: is composed of type nodes and α nodes. Type node denotes the type of condition, and α node denotes constraint condition, both of them are single input ones. The α network is used to store the rule condition instances. The root node receives the fact property of an object, and thus judges what kind of type node the constraint condition is. Each kind of type node corresponds to different α node. Moreover, fact property enters into the β network to match the β node according to condition type and constraint condition. Each α node represents a rule condition. That is, each α node stores the constraint condition for rule selection [24].
- β network: is composed of β nodes, which are double input nodes. β node is used to compare two objects and their fields. The type of objects compared may be the same or different. The two inputs of a double input node are named left input and right input, respectively. Generally, left input is a group of object lists, and right input is a certain object. The result of α node can be added into the β node pattern. Then we check whether there is a fact that meets the condition or not in the other input set. If yes, it enters the corresponding β node pattern. Otherwise, it can be regarded as an action execution node, and the corresponding action will be executed on an action execution node.
- Action node: including a series of actions. Each action node has its corresponding action. Each action node has its own unique constraint conditions. Because each kind of constraint condition corresponds to different actions, the number of action nodes is unknown.

**Figure 7:**
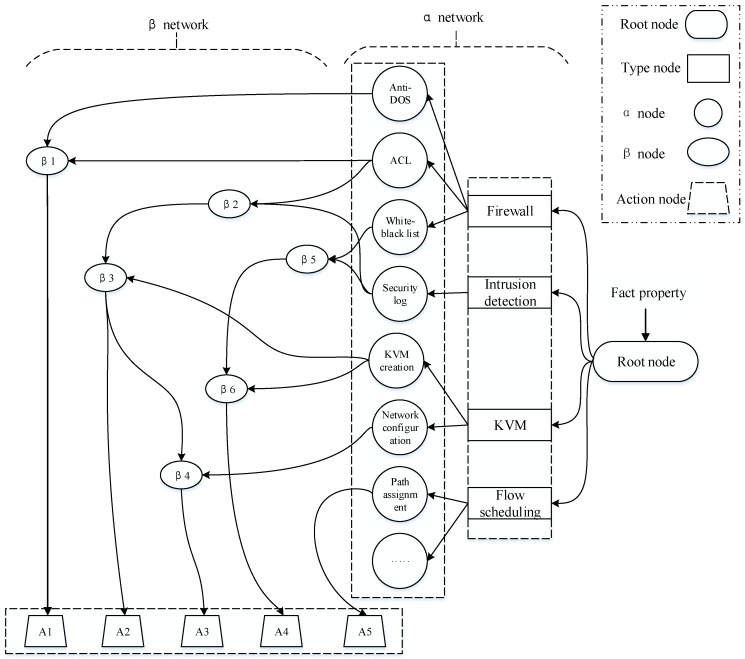
Optimized security rule composition network.

The dynamic security service construction optimized algorithm for SDN takes the ideas from RETE algorithm. The rules combined can be compiled into a RETE network. Four types of security services are regarded as type nodes, and the security meta-functions for each kind of service are regarded as the α node. Moreover, the composition results from different security meta-functions are regarded as β nodes, and the specific actions for composition results are regarded as action execution nodes. The following two points should be noted:

- In our solution, type nodes and α network can be pre-complied, however, β network can be formed by constantly composition.
- In contrast to the traditional RETE network, there may exist the combination among α nodes under the same type node. Hence, the structure of the optimized security rule composition network is illustrated in Figure 7.

In the optimized security rule composition network, all the meta-functions, as rule composition conditions, constitute the α networks. Different combinations of these meta-functions constitute different security services. Different security services correspond to different β nodes, and different security services combined execute different actions. For example, when a service requirement needs meta-functions Anti-DOS and ACL in the library, it can directly enter into the $\beta_{1}$ node pattern and then execute action $A_{1}$. For another service requirement, it may require meta-functions ACL, security log as well as KVM creation, which can be combined as the $\beta_{3}$ node pattern and execute action $A_{2}$. Particularly, if a new service requirement, which adds meta-function network configuration on the basis of $\beta_{3}$ is proposed, then the $\beta_{4}$ node pattern is created by combining $\beta_{3}$ and network configuration, and thus execute action $A_{3}$. Because the security rule composition network is pre-compiled, the combination time is greatly reduced without traversing the whole rule database.

## 5. Emulation & Analysis

**Figure 8:**
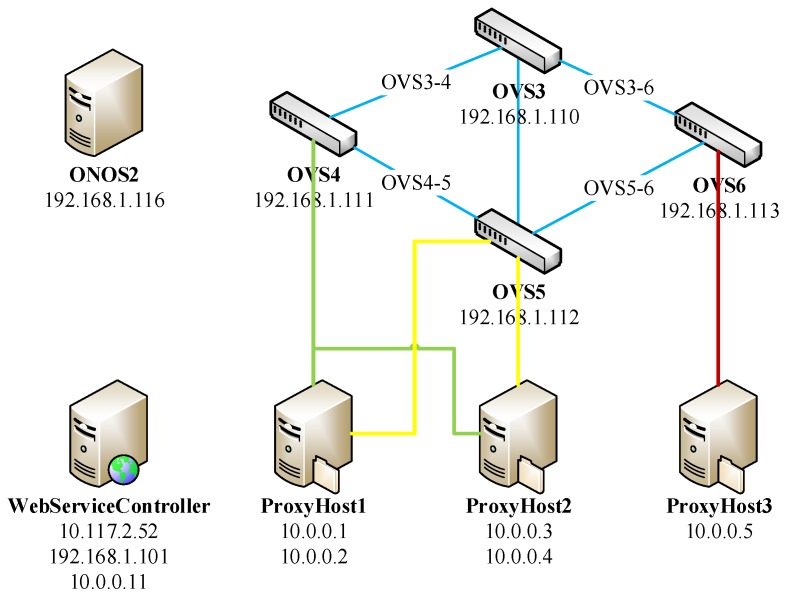
Network topology.

Currently, there are two mature cloud platforms [25]: VMware and OpenStack, which can support the construction of SDN environment. Considering the flexible requirement and high demand on migration of VMs, this paper selects an OpenStack platform, which can be deployed on three servers. Server 1 can be configured as the control node, and Server 2 and Server 3 can be configured as data nodes. We put the security service orchestration center on WebServiceController in order to realize decoupling with Open Network Operating System (ONOS) controller. The whole network topology can be illustrated in Figure 8. This network topology includes an ONOS controller with IP address 192.168.1.116, a WebService controller with IP address 192.168.1.101 used to communicate with ONOS controller and 10.0.0.11 used to communicate with ProxyHosts, 4 OVSes are on network segment 192.168.1.110∼192.168.1.113 and 3 ProxyHosts are on network segment 10.0.0.1∼10.0.0.5, respectively.

The configuration requirements of VMs in the network topology are listed in Table 3.

### 5.1. Function Test

In this part, the service requirement can be described as follows. ProxyHost 10.0.0.1 configures itself with firewall in order to prevent DOS attack. We set the ProxyHost 10.0.0.3 to blacklist on ProxyHost 10.0.0.1. An intrusion detection system is deployed, and all the data packets that reach ProxyHost 10.0.0.1 are assigned to pass OVS5 (192.168.1.112) only but not OVS3 (192.168.1.110).

**Figure 9:**
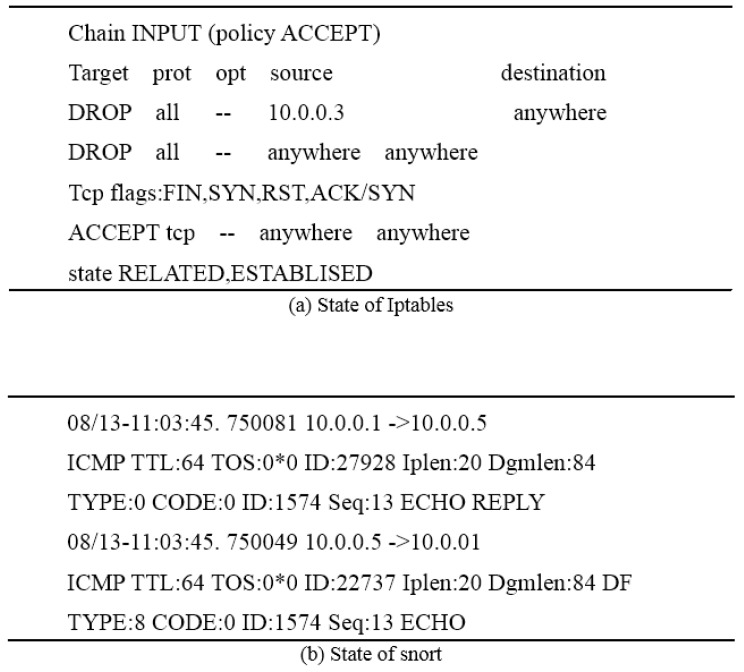
Configuration result of Firewall.

A service requirement can be submitted on the client side. From the configuration result of the Firewall shown in Figure 9, we can obtain the result that (1) all the data packets from ProxyHost 10.0.0.3 were dropped; and (2) the attack for SYN was also dropped.

**Figure 10:**
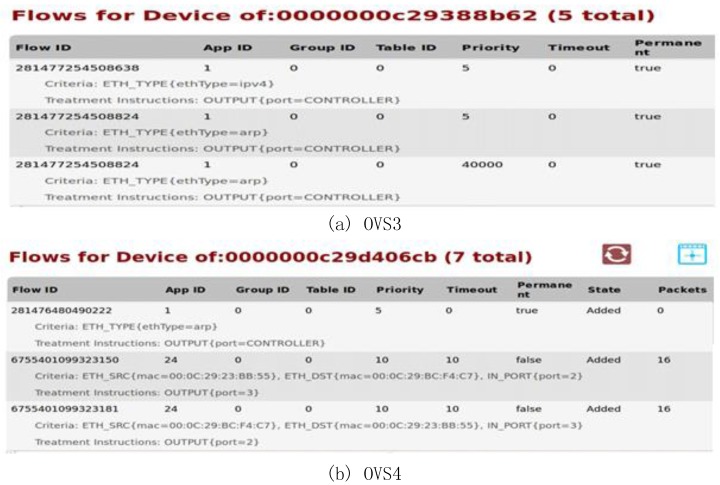

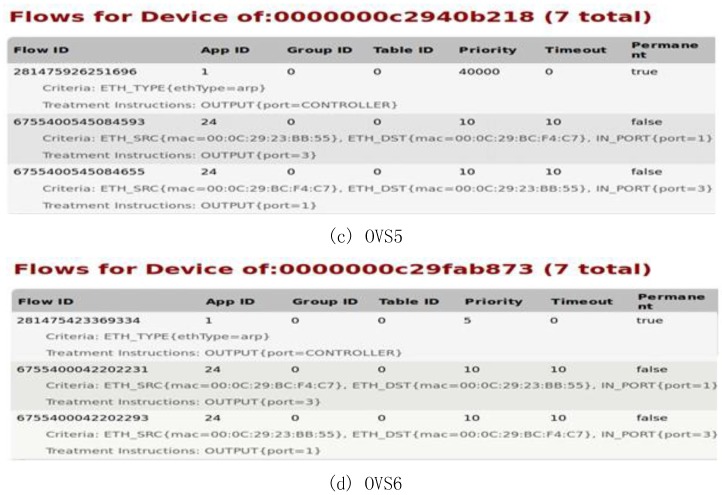
Flow charts of (**a**) OVS3; (**b**) OVS4; (**c**) OVS5 and (**d**) OVS6.

By looking at the flow information given in Figure 10, we can find that all the OVSes (OVS4, OVS5 and OVS6) have flow information without including OVS3. It shows all the data packets that reach the ProxyHost10.0.0.1 cannot pass OVS3 (192.168.1.110).

The results mentioned above show that the firewall service, intrusion detection service, flow scheduling service and KVM service can functionally achieve the design requirements.

We can conclude that, compared to VSA and SDS, our security protection architecture design has the following two characteristics:

- From the testing results, the issuing and implementing cannot be controlled by SDN in our architecture. That is, the security protection of this architecture is decoupled from the SDN controller. Even if the SDN controller itself is attacked, the network still has the ability of security protection.
- From the protection results, our architecture can achieve security protection by fully combining the advantages of VSA and SDS. Specifically, security can be embedded into SDN network by traditional security device virtualization, and the control plane and data plane are it separated and reconstructed so as to achieve modularity and reusability.

### 5.2. Performance Test

#### 5.2.1. Test on the Rule-Combining Time for Single User

**Figure 11:**
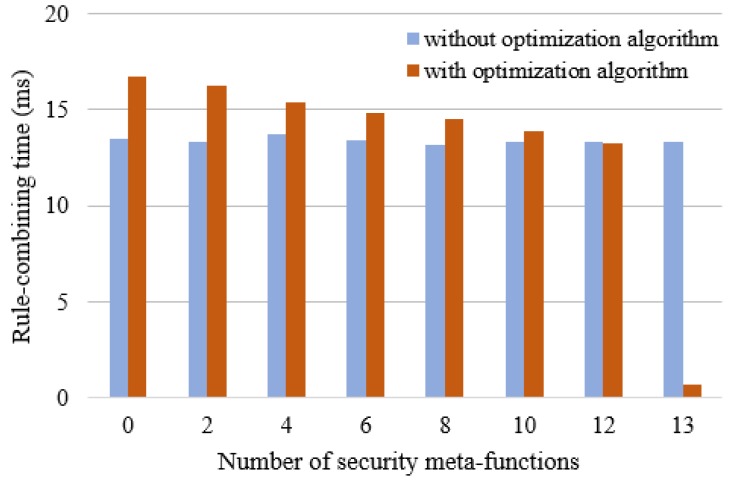
The rule-combining time comparison with and without using optimization algorithm for single user.

For the rule-combining time, the influence of the dynamic security service assembly algorithm for SDN mainly concentrates on the fact that the corresponding rules can be quickly searched without traversing the whole rule database. In our experiment, the number of security meta-functions contained in the state table be 13. For each composition, the number of security meta-functions recorded in β network gradually increases, until all the security meta-functions are involved. The rule-combining time comparison with and without using the optimization algorithm for single user can be illustrated in Figure 11.

The experimental results show that the rule-combining time without using the optimization algorithm is largely unchanged mainly because is traverses the whole rule database each time. The rule-combining time with using optimization algorithm is greater than that without using optimization algorithm at the beginning. For example, when the number of security meta-functions is 2, the rule-combining time with and without using optimization algorithm are 16.27 ms and 13.32 ms, respectively. This is because the optimization algorithm firstly needs to construct the RETE rule network. As the number of security meta-functions recorded (that is, the shared nodes) in β network increases, the rule-combining time decreases. For instance, when the number of security meta-functions is 12, the rule-combining time with and without using the optimization algorithm are 13.26 ms and 13.30 ms, respectively. Once 13 security meta-functions are involved in the β network, the rule-combining time with using optimization algorithm drastically reduces to 0.66 ms.

#### 5.2.2. Test on the Rule-Combining Time for Multiple Users

**Figure 12:**
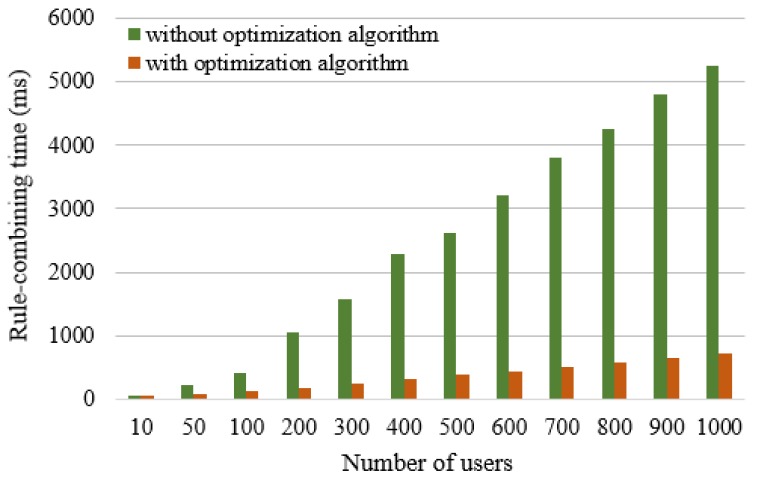
The rule-combining time comparison with and without using optimization algorithm for multiple users.

Next, we discuss the case that multiple users request security service. The rule-combining time comparison with and without using the optimization algorithm for multiple users can be illustrated in Figure 12.

The experimental results show that, with the increase of the number of users, the rule-combining time based on an expert system will increase about 500 ms for every 100 user requests increase. After the optimization algorithm is used, the combination time is greatly shortened. With the increase of the shared nodes in the RETE network, the growth of the combining time gradually tends to be gentle. For example, when the number of users is 600, the rule-combining time with and without using the optimization algorithm are 443 ms and 3212.5 ms. When the number of users is 800, the rule-combining time with and without using optimization algorithm are 580.2 ms and 4246.8 ms, respectively.

### 5.3. Comparison

**Figure 13:**
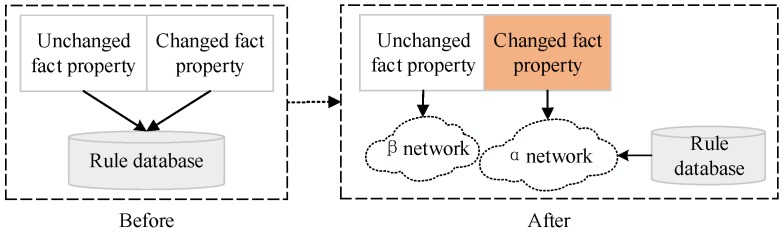
Comparison between two algorithms.

We can obtain the result that the proposed dynamic security service composition algorithm is a kind of efficient pattern matching algorithm. The rule matching process will be repeated without using the optimization algorithm. Generally, the fact property contains two parts: a changed and unchanged one, as is shown in Figure 13.

The fact property will be modified dynamically in order to meet all the requirements. Once the fact property changes, the traversal for rule database will happen. It has to prolong the rule-combining time and affect the user experience. In contrast, the meta-functions can be recorded and stored by using the optimization algorithm. Different security services correspond to the same security meta-functions, and the share among β nodes can be achieved. Under the condition of a new requirement, we just check whether the fact property in the new request changes or not. If yes, what we need to do is add or delete the corresponding service composition(s) in the α network compiled by the rule database.

Hence, our security service composition algorithm can make full use of the structural redundancy and similarity of rule to achieve intermediate state storage as well as multiple nodes sharing, and thus significantly improve the efficiency of the service composition.

## 6. Conclusions

In this paper, a novel security architecture is proposed to solve the security problem in Software Defined Networks. We design a security service orchestration center in the control plane of SDN, which physically decouples from the SDN controller. Virtualization technology is used to construct a security meta-function library, and a dynamic security service composition construction algorithm is proposed. Particularly, to improve the efficiency of the rule-combining method, the RETE algorithm is introduced. Convincing experimental results show that our solutions can effectively provide users with security protection and improve the efficiency of the service composition.

In our future work, we plan to investigate more flexible security service composition model for different types of users in order to satisfy their corresponding knowledge on SDN security.

## Figures and Tables

**Table 1:** Four types of security meta-functions included.

Type	Name	Description
Firewall	ACL_FUNCTION	ACL access control
	DOS_FUNCTION	Anti-DOS attack
	WBLIST_FUNCTION	White-black list control
	CON_FUNCTION	Policy configuration
Intrusion detection	NETDEC_FUNCTION	Network detection
	LOG_FUNCTION	Security log
	RULE_FUNCTION	Rule configuration
	WAR_FUNCTION	Real-time warning
KVM	KVMCRE_FUNCTION	KVM creation
	NETCON_FUNCTION	Network configuration
	KVMDEL_FUNCTION	KVM deletion
Flow scheduling	PATHDES_FUNCTION	Path assignment
	PATHLIM_FUNCTION	Limited path

**Table 2:** Some parameters used in the process of forward reasoning.

Parameter	Meaning
$T_{i}$	state
$e_{i}$	condition
$R_{i}$	rule
$R_{i}\overset{x_{i}}{\to }R_{j}$	rely relationship
$l_{i}$	priority

**Table 3:** The configuration requirements of VMs in the network topology.

Parameter	Configuration
OS	Ubuntu 14.04
OS Type	64 bit
CPU Type	Intel Core i5-3210M CPU@2.5GHz*4
Memory	1.9 G
Disk	31.3 GB

## Abbreviations

The following abbreviations are used in this manuscript:

ACL	Access Control Layer
BRAS	Broadband Remote Access Server
DOS	Denial of Service
JSON	JavaScript Object Notation
KVM	Kernel-based Virtual Machine
NE	Network Element
NFV	Network Function Virtualization
ONOS	Open Network Operating System
OVS	Open VSwitch
SDN	Software Defined Network
VM	Virtual Machine
